# Supplementary material for: Diversification of ergot alkaloids and heritable fungal symbionts in morning glories
Source: Commun Biol. 2021 Dec 6;4:1362. doi: 10.1038/s42003-021-02870-z (PMC8648897; doi:10.1038/s42003-021-02870-z)
Supplement: Supplementary file 2 — Description of Additional Supplementary Files [file 42003_2021_2870_MOESM2_ESM.pdf]

Description of Additional Supplementary Files

File name: Supplementary Data 1
Description: Sampling information and results. Naming authority and geographic information were collected from the World Checklist of Selected Families at https://wcsp.science.kew.org.

File name: Supplementary Data 2
Description: Accession information of all sequences included in the ITS phylogeny.

File name: Supplementary Data 3
Description: ITS phylogeny of morning glories with EAs. ITS phylogeny adapted from Munoz et al.[27] colorized with our EA data. Orange species are EA- and blue species are EA+.

File name: Supplementary Data 4
Description: All herbarium specimens used in the study. Source data for Figure 1.

File name: Supplementary Data 5
Description: *Ipomoea, Argyrei, Stictocardia,* and *Turbina* occurence data from GBIF. Source data for Figure 1.

File name: Supplementary Data 6
Description: RaxML phylogeny. Source data for Figure 2, 3, and 4.

File name: Supplementary Data 7
Description: Metadata for species in the phylogeny. Source data for Figure 2.

File name: Supplementary Data 8
Description: Ergot alkaloid data for species in the phylogeny. Source data for Figure 4.

File name: Supplementary Data 9
Description: Source data for Figure 5.
