# Supplementary material for: Diversification of ergot alkaloids and heritable fungal symbionts in morning glories
Source: Commun Biol. 2021 Dec 6;4:1362. doi: 10.1038/s42003-021-02870-z (PMC8648897; doi:10.1038/s42003-021-02870-z)
Supplement: Supplementary file 12 — Reporting Summary [file 42003_2021_2870_MOESM12_ESM.pdf]

nature portfolio

Corresponding author(s): Keith Clay
Last updated by author(s): Oct 7, 2021

Reporting Summary

Nature Portfolio wishes to improve the reproducibility of the work that we publish. This form provides structure for consistency and transparency in reporting. For further information on Nature Portfolio policies, see our Editorial Policies and the Editorial Policy Checklist.

Statistics

For all statistical analyses, confirm that the following items are present in the figure legend, table legend, main text, or Methods section.

n/a	Confirmed	
☐	☒	The exact sample size (n) for each experimental group/condition, given as a discrete number and unit of measurement
☐	☒	A statement on whether measurements were taken from distinct samples or whether the same sample was measured repeatedly
☐	☒	The statistical test(s) used AND whether they are one- or two-sided<br>*Only common tests should be described solely by name; describe more complex techniques in the Methods section.*
☒	☐	A description of all covariates tested
☐	☒	A description of any assumptions or corrections, such as tests of normality and adjustment for multiple comparisons
☐	☒	A full description of the statistical parameters including central tendency (e.g. means) or other basic estimates (e.g. regression coefficient) AND variation (e.g. standard deviation) or associated estimates of uncertainty (e.g. confidence intervals)
☐	☒	For null hypothesis testing, the test statistic (e.g. F, t, r) with confidence intervals, effect sizes, degrees of freedom and P value noted<br>*Give P values as exact values whenever suitable.*
☒	☐	For Bayesian analysis, information on the choice of priors and Markov chain Monte Carlo settings
☒	☐	For hierarchical and complex designs, identification of the appropriate level for tests and full reporting of outcomes
☐	☒	Estimates of effect sizes (e.g. Cohen's d, Pearson's r), indicating how they were calculated

Our web collection on statistics for biologists contains articles on many of the points above.

Software and code

Policy information about availability of computer code

Data collection	no software was used in data collection
Data analysis	R was used to perform basic statistical analysis. Other analyses was done using R packages "ips", "ape", "geiger", "caper", "phytools", "phylolm" and "vegan". Please see the Methods section for complete descriptions.

For manuscripts utilizing custom algorithms or software that are central to the research but not yet described in published literature, software must be made available to editors and reviewers. We strongly encourage code deposition in a community repository (e.g. GitHub). See the Nature Portfolio guidelines for submitting code & software for further information.

Data

Policy information about availability of data

All manuscripts must include a data availability statement. This statement should provide the following information, where applicable:
- Accession codes, unique identifiers, or web links for publicly available datasets
- A description of any restrictions on data availability
- For clinical datasets or third party data, please ensure that the statement adheres to our policy

All data is included in the manuscript or can be found on Figshare at https://doi.org/10.6084/m9.figshare.14749512. All accession codes for GenBank sequences used are included in our supplementary data.

Field-specific reporting

Please select the one below that is the best fit for your research. If you are not sure, read the appropriate sections before making your selection.

☐ Life sciences ☐ Behavioural & social sciences ☒ Ecological, evolutionary & environmental sciences

For a reference copy of the document with all sections, see nature.com/documents/nr-reporting-summary-flat.pdf

Ecological, evolutionary & environmental sciences study design

All studies must disclose on these points even when the disclosure is negative.

Study description	This study was based on chemical analysis of seeds from morning glory species (Convolvulaceae) for ergot alkaloids, which are indicative of symbiosis with Periglandula fungi, the source of the ergot alkaloids. Seeds were primarily obtained from historical specimens from 3 herbaria plus field collections of seeds from two common species in the US. Chemical data were mapped onto a phylogenetic tree and grouped by a major plant life history trait (seed size) to provide novel insights into the evolution of a speciose plant family, the distribution of fungal symbiosis and plant-fungal chemical coevolution.
Research sample	Mature seeds from herbarium sheets plus seeds from field collections.
Sampling strategy	Seeds were primarily sampled from historical herbarium specimens with permission. Most specimens do not have mature seeds so we sampled seeds from only a subset of possible species. We also made field collections of two common species in the US from 20-30 locations/populations per species.
Data collection	Seeds were pulverized, extracted in methanol and extracts were analyzed from ergot alkaloids by the Panaccione lab at WVU. Chemical data were recorded on spreadsheet associated with sample number.
Timing and spatial scale	Herbarium specimens were sampled in 2014-2016 but the historical herbarium specimens themselves were collected from 5-120 years ago. Most samples were 20-40 years old and came from all continents except Europe and Antarctica as illustrated in Figure 1. Field collected seeds were obtained in 2014-2015 from the Great Plains region of the US (Ipomoea leptophylla) and Florida (Ipomoea pes-caprae).
Data exclusions	All alkaloid data were utilized with no exclusions.
Reproducibility	Reproducibility was tested by testing multiple independent samples per species for ergot alkaloids. For some species where only a single sample existed, we were not able to test multiple samples as described in the text of the manuscript.
Randomization	Not applicable to this study.
Blinding	Seed extracts were sent to the Panaccione lab blind with just an arbitrary number for identification and no information about species, age or location.

Did the study involve field work? ☒ Yes ☐ No

Field work, collection and transport

Field conditions	Seed collections were made across several days and from 20-30 sites/populations for each species. Ergot alkaloid content per seed is not sensitive to short term environmental variations.
Location	The location and date of collection of all samples analyzed in this study are provided in the Supplemental Materials. For some herbarium specimens it was not possible to obtain exact location data from the information on the herbarium label so we used the midpoint of the country, territory or county in those cases.
Access & import/export	Seeds were obtained from one US herbarium, one Netherlands herbarium and one Australian herbarium. All field collection of seeds were from the US on public lands where no permits were required. Seeds samples for the Netherlands were pulverized before transport to the US so no permits were required. Seeds were shipped from Australia to the Indiana University herbarium under international regulations for exchange of herbarium samples.
Disturbance	Less than 50% of mature seeds were collected from any one herbarium specimen with permission and noted on the herbarium label. Field collections were made along publically accessible roadside right-of-ways and consisted of only 10-20 seeds out of 10,000 to 100,000 being produced at the particular population.

Reporting for specific materials, systems and methods

We require information from authors about some types of materials, experimental systems and methods used in many studies. Here, indicate whether each material, system or method listed is relevant to your study. If you are not sure if a list item applies to your research, read the appropriate section before selecting a response.

Materials & experimental systems

n/a	Involved in the study
☒	☐ Antibodies
☒	☐ Eukaryotic cell lines
☒	☐ Palaeontology and archaeology
☒	☐ Animals and other organisms
☒	☐ Human research participants
☒	☐ Clinical data
☒	☐ Dual use research of concern

Methods

n/a	Involved in the study
☒	☐ ChIP-seq
☒	☐ Flow cytometry
☒	☐ MRI-based neuroimaging
